# Human Lung Organoids—A Novel Experimental and Precision Medicine Approach

**DOI:** 10.3390/cells12162067

**Published:** 2023-08-15

**Authors:** Laura Kühl, Pauline Graichen, Nele von Daacke, Anne Mende, Malgorzata Wygrecka, Daniel P. Potaczek, Sarah Miethe, Holger Garn

**Affiliations:** 1 Translational Inflammation Research Division & Core Facility for Single Cell Multiomics, Medical Faculty, Philipps University of Marburg, Member of the German Center for Lung Research (DZL) and the Universities of Giessen and Marburg Lung Center, 35043 Marburg, Germany; kuehll@students.uni-marburg.de (L.K.); graichen@students.uni-marburg.de (P.G.); daacken@students.uni-marburg.de (N.v.D.); mende@students.uni-marburg.de (A.M.); potaczek@staff.uni-marburg.de (D.P.P.); 2 Center for Infection and Genomics of the Lung (CIGL), Universities of Giessen and Marburg Lung Center (UGMLC), 35392 Giessen, Germany; malgorzata.wygrecka@innere.med.uni-giessen.de; 3 Institute of Lung Health, German Center for Lung Research (DZL), 35392 Giessen, Germany; 4 CSL Behring Innovation GmbH, 35041 Marburg, Germany; 5 Bioscientia MVZ Labor Mittelhessen GmbH, 35394 Giessen, Germany

**Keywords:** disease models, lung epithelium, airways, organoids, personalized medicine, translational research

## Abstract

The global burden of respiratory diseases is very high and still on the rise, prompting the need for accurate models for basic and translational research. Several model systems are currently available ranging from simple airway cell cultures to complex tissue-engineered lungs. In recent years, human lung organoids have been established as highly transferrable three-dimensional in vitro model systems for lung research. For acute infectious and chronic inflammatory diseases as well as lung cancer, human lung organoids have opened possibilities for precise in vitro research and a deeper understanding of mechanisms underlying lung injury and regeneration. Human lung organoids from induced pluripotent stem cells or from adult stem cells of patients’ samples introduce tools for understanding developmental processes and personalized medicine approaches. When further state-of-the-art technologies and protocols come into use, the full potential of human lung organoids can be harnessed. High-throughput assays in drug development, gene therapy, and organoid transplantation are current applications of organoids in translational research. In this review, we emphasize novel approaches in translational and personalized medicine in lung research focusing on the use of human lung organoids.

## 1. Introduction

The global incidence of respiratory diseases is high and still rising, even without the recent coronavirus disease 2019 (COVID-19) pandemic and its associated morbidity and mortality [1,2]. Infectious lung diseases are the third-leading cause of premature mortality. Among non-communicable diseases, chronic respiratory disorders significantly contribute to global morbidity. In 2017, 544.9 million people worldwide suffered from chronic respiratory diseases which also ranked third as global cause of death [3]. Lung cancer is the leading cause of cancer-related mortality worldwide [4]. The health burden of lung diseases is expected to rise even higher in the next 20 years: Lower respiratory tract infections are predicted to increase prompting serious implications for global health resources regarding the aging population. Premature mortality and deaths due to chronic respiratory diseases are projected to rise with tobacco smoke exposure and ambient air pollution among the five most important risk factors contributing to risk-attributable premature mortality [5].

To fully comprehend the underlying mechanisms of health and disease in the respiratory system, research models need to become more accurate with regard to the representation of physiological and pathophysiological processes. Models should mirror specific aspects of the in vivo situation as precisely as possible to grant transferability for clinical studies, which often require higher model complexity. Current models such as in vitro generated human lung organoids, which represent three-dimensional (3D), multi-cell types-containing, and self-assembling structures, offer new insights into tissue-specific intercellular communication processes mimicking parenchymal interactions. A better understanding of lung physiology, injury, and repair is necessary to develop suitable therapeutic approaches for various lung diseases. Therefore, 3D organoid cultures may be used to study underlying epithelial mechanisms. Further, the development of expandable organoids grown from stem cells or patient tissue samples provides a method for high-throughput screening of drugs [6]. This review aims to provide a perspective on the potential uses of lung organoids for translational and personalized medicine.

## 2. Lung Epithelium

**Figure 1:**
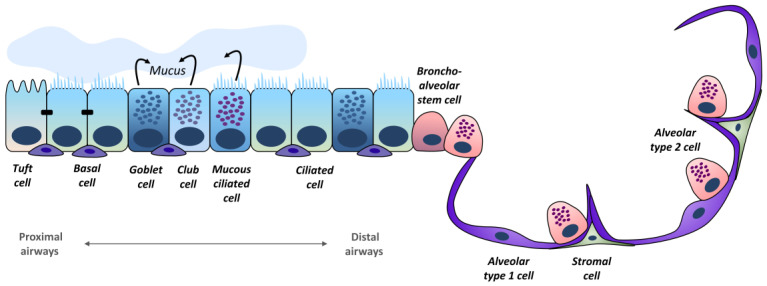
Cell components of the respiratory system. Airways are formed by a pseudostratified epithelium composed of diverse basal, ciliated, and secretory cells. The alveoli are lined by squamous alveolar type 1 cells and cuboidal alveolar type 2 cells (adapted from Barkauskas et al. [8]).

The lower respiratory tract consists of the trachea, bronchi, and bronchioli, representing the lower conducting airways and the alveoli. The conducting airways are responsible for mucociliary clearance and consist mainly of basal, ciliated, club, and goblet cells (Figure 1). Gas exchange takes place in the alveoli which are built of alveolar type 1 (AT1) and alveolar type 2 (AT2) cells [7]. 

The epithelial composition changes from proximal to distal airways. Cell populations in the tracheal and intermediate bronchi are quite similar, as they contain mainly basal cells and fewer differentiated cell types. Distal airways have a higher percentage of secretory and multiciliated cells [9]. Basal cells are the stem cells of the bronchial epithelium and constitute about one-third of cells in human airways [9]. Subsets of basal cells in different states have been described, including proliferating or immuno-activated subgroups [7]. They play an important role in the regulation of physiological and pathological differentiating processes because of their ability to differentiate into diverse secretory and ciliated cells. In turn, goblet and club cells produce mucus which is essential in lung physiology to protect the mucous membranes. Yet, under pathological conditions goblet cell hyperplasia induces chronic mucus hyperproduction resulting in an impaired mucus clearance [10,11,12]. Ciliated cells are needed for mucociliary clearance and play a part in cytoprotection and fluid reabsorption [7,13]. Mucous ciliated cells represent a transitional cell type expressing both mucous and ciliated markers [7]. Present in proximal airways, tuft cells, and their chemosensory function play a role in the regulation of respiration, while their production of pro-inflammatory cytokines, such as interleukin (IL)-25, contributes to type two inflammatory processes [14,15]. Bronchoalveolar stem cells (BASC) are located at bronchioalveolar–duct junctions. Stable during lung homeostasis, upon lung injury they contribute to lung regeneration as they are able to differentiate into club and ciliated cells and thereby regenerate the terminal bronchiole [16]. Rare cell types in the airway epithelium are ionocytes and pulmonary neuroendocrine cells (PNEC). Ionocytes comprise only 0.5–1% of airway epithelial cells and are responsible for the majority of cystic fibrosis (CF) transmembrane conductance regulator (CFTR)-activity in the lung epithelium [17]. PNEC have hormonal and immune functions: They react to environmental stimuli and secrete Calcitonin Gene-Related Peptide (CGRP) which induces goblet cell hyperplasia [18]. While the airway epithelium is composed of a multiplicity of different cell types, the epithelium of the air sacs consists of mainly two types of cells: AT1 and AT2 cells. AT1 cells cover 95% of the alveolar surface and are responsible for gas exchange, while AT2 cells produce surfactant and have stem cell capacity [19,20]. Stromal cells contribute to matrix biosynthesis while also carrying out organ-specific tasks: Alveolar fibroblasts play a role in immune defense mechanisms, whereas myofibroblasts are responsible for the maintenance of the lung scaffold [21]. There are noticeable structural, cellular, and molecular differences between human lungs and the respiratory system of experimental animals, specifically rodents, which needs to be considered in translational approaches (see also Section 3.7).

## 3. Human Cell-/Tissue-Based Model Systems

**Figure 2:**
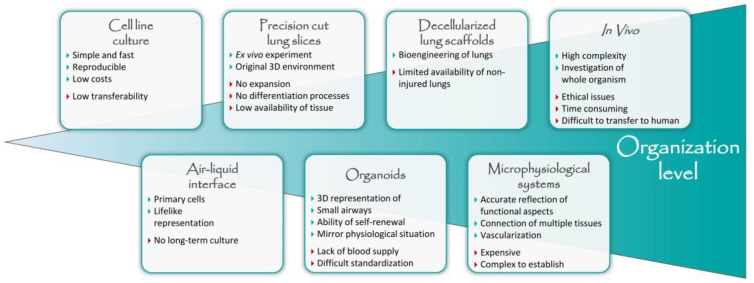
Diversity of currently available model systems for lung research.

The high complexity of the lung anatomy and physiology results in the exceptional need for well-developed model systems and, therefore, it is important to carefully choose which model system best meets the needs of a given research question. In Figure 2 a diversity of currently available model systems relevant for translational lung research are depicted, which will be discussed in more detail within this chapter.

### 3.1. 2D Cell Cultures

Simple immortalized cell line cultures are the easiest and fastest model systems, making their application common in lung research. These are typically used to answer basic research questions related to airway cell pathophysiology [22]. Commonly used cell lines in lung research are for instance BEAS2B, Calu-3, or IB3-1 cells (all derived from bronchial epithelium) or A549 (derived from a lung carcinoma representing alveolar epithelium) [23,24,25,26]. They are used to study basic pathomechanisms in lung diseases such as chronic obstructive pulmonary disease (COPD), cystic fibrosis (CF), or host–virus interactions in infectious diseases like COVID-19 [27,28]. The advantages of 2D cell culture systems are numerous, including reproducibility, easy handling, low costs, and long-term culturing [29]. However, these advantages are achieved by use of immortalized cell lines, which may have quite different properties and genetic backgrounds than primary cells [30]. Therefore, they cannot mirror the in vivo situation properly. Another disadvantage of 2D cell culture systems is the non-physiologically uniform cell environment [30]. This includes the lack of diverse cell–cell and cell–matrix interactions as well as missing cellular communication between heterogenic cell types [31]. In conclusion, immortalized 2D cell cultures have several advantages, especially for gaining preliminary understanding; however, they do not accurately represent the in vivo situation. Simple 2D cell cultures may also be established with primary cells such as normal human bronchial (epithelial) cells (NHBCs). However, such cells are difficult to obtain, they are expensive, and short-lived and thus more likely to be used in more complex model systems as outlined below.

### 3.2. Air–Liquid Interface Cultures

Air–liquid interface (ALI) cultures have been developed to increase the complexity of cell culture model systems as they better represent physiological conditions and involve cellular differentiation processes. These culture systems can be established with primary airway epithelial cells or cell lines such as BEAS2B or A549, which grow as pseudostratified epithelium with a mucociliary phenotype [32,33]. To achieve this, cells are seeded on nano-porous membranes and placed on inserts into wells of a cell culture plate. After initial proliferation, the medium on the apical (upper) side of the membrane is withdrawn, resulting in cells directly exposed to air (air-lift). Thus, only the basal (lower) side of the cell layer remains in contact to the nutrient supply in the medium, the epithelial integrity within the ALI culture is well maintained by tight junctions [34,35]. This results in a differentiated and polarized epithelial structure reflecting the in vivo situation more precisely than the above-described simple cell culture systems. The development of primary cells into a pseudostratified epithelium results in a reasonable reflection of the airway epithelium, including different cell types such as basal, ciliated, and secretory cells. Fascinatingly, mucus production and cilia movement may be observed in this cell culture system [34]. Due to the compartmentalization, ALI cultures offer the opportunity to stimulate cells physiologically from either the apical or the basal side. Furthermore, co-culturing of the epithelial cells e.g., with immune cells, endothelial cells, or fibroblasts is possible and further enhances the complexity of this model system [36,37]. Using primary airway cells, cell availability might limit research options. However, the main drawback of ALI cultures is that they cannot be maintained for an extended time period, as cells will eventually die following final differentiation [28]. Hence, it is difficult to use ALI cultures for high-throughput or long-term assays.

Overall, compared to simple 2D cell culture models, ALI cultures are far more analogous to the in vivo situation and may be used to analyze drug delivery mechanisms, differentiation processes, and mechanisms of development of and interference with epithelial barrier integrity.

### 3.3. Precision Cut Lung Slices

Precision cut lung slices (PCLS) are thin slices of tissue taken from freshly obtained human or animal lung samples, which can be collected from surgical sections [38,39]. The primary advantage of PCLS is, that they preserve the original 3D environment, hence the architecture and mechanical properties as well as the cellular composition of the tissue is maintained, including presence of various cell types as well as established cell–cell and cell–matrix interactions [38]. This, in turn, enables the analysis of both functional and microenvironmental characteristics of healthy and diseased lungs [31]. Further, this experimental system is suitable for modeling inflammatory disease conditions by showing key features of inflammatory exacerbations upon ex vivo pathogen stimulation [40,41]. Other applications of PCLS are ex vivo investigations of disease development and progression, as well as screening for potential drugs [42,43]. Insights into primary toxicity as well as the molecular mode of action of potential drug candidates can be gained. As an example, mouse and human PCLS were used to investigate anti-fibrotic drug effects of Nintedanib and Pirfenidone on lung epithelial cell functions [42]. PCLS can be generated from healthy and diseased donors and thus also exacerbation processes can be analyzed [38,44]. Studies modelling chronic lung diseases such as idiopathic pulmonary fibrosis (IPF) and COPD have been performed using PCLS from respective mouse models [45,46]. Certainly, PCLS from mouse models are rather easy to obtain; however, these models may not accurately represent human lung diseases. In contrast, the availability of respective human tissue samples is a limiting factor. Another disadvantage of PCLS is that they can only be cultured for a rather short period of two weeks with ongoing unphysiological degradation processes over time [38]. In addition, the number of slices, which can be cut from one tissue sample of varying size is limited as well. Therefore, the standardization of PCLS approaches remains difficult [31,38]. For immunological studies, autologous inflammatory components might have to be supplemented as immune cells are less abundant in tissue slices [38].

Concluding, PCLS represents a highly complex ex vivo model system for a variety of scientific questions; nevertheless, limiting factors such as tissue availability and maintenance need to be considered.

### 3.4. Organoids

Organoids are 3D cell culture systems derived from adult stem cells or pluripotent stem cells. Under well-defined culture conditions, these cells have the ability to proliferate and differentiate into various epithelial cell types quite similar to in vivo. During this process, the cells self-assemble into spherical structures [30,47]. Lung organoids mirror the physiological in vivo situation in their cellular composition and functionality both similar to the situation within the organ of origin, including cell-cell and cell-matrix interactions. Organoids are able to self-renewal as the regenerative capacity of the stem cells is maintained for a longer period of time, which is of great advantage for the investigation of organ development, differentiation, and repair processes in vitro [22,31,47]. Further characteristics and translational applications of human lung organoids will be discussed in more detail in Section 4, Section 5 and Section 6.

### 3.5. Decellularized Lung Scaffolds

Another new approach to highly complex lung model systems is decellularized lung scaffolds. To obtain these scaffolds, lung tissue is decellularized using physical, chemical, and/or enzymatic treatments. The remaining extracellular matrix is reseeded with induced pluripotent stem cells (iPSC). The matrix scaffold provides both structural and biochemical support [48]. Various detergent- and freezing-based techniques were established; however, the matrix could not always be kept entirely intact. Additionally, these methods have not achieved complete recellularization so far [49]. The heterogeneity of human donor tissue creates additional challenges. Ohata and Ott [48] stated further that donor lungs could be injured either from death or donation procedures, limiting the availability of non-injured lung tissues [50]. Additionally, the bioengineered lungs are influenced by the age or pathologic phenotype of donor lung scaffolds including patients’ cell ability to adhere, proliferate, or even survive. As such, it has been observed that abnormal recellularization of scaffolds obtained from diseased lungs may lead to an unintended pathological phenotype which may interfere with the results obtained for donor cells used in the recellularization process. In turn, these characteristics may specifically be used as a model system to analyze cell–matrix interactions and are, therefore, an excellent tool in lung regeneration medicine [50].

Decellularized lung scaffolds may be used in the future to bioengineer new lungs, potentially replacing lacking donor lungs or being used as a highly complex model system in research. However, several challenges still remain to be solved as outlined above.

### 3.6. Microphysiological Systems

While searching for new and suitable model systems with a high complexity and high potential to reflect the in vivo situation, one reads often about microphysiological systems. The definition of such systems differs depending on the source. Some authors even include organoids in this definition, whilst others use microphysiological systems as a synonym for organs-on-a-chip. Microphysiological systems should feature the main anatomical and physiological, hence structural and functional, characteristics of the human organ [22]. In organs-on-a-chip model systems, the goal is to culture a miniaturized tissue unit in a way that most accurately reflects functional aspects of the whole structure [51,52]. Multiple layers of cells in a lung-on-chip model and the presence of dynamic mechanical forces induced by the fluidic flow resemble the breathing organ. A great advantage of the organs-on-a-chip model is that multiple tissue systems can be connected in a microfluidic channel allowing them to communicate [31]. This bridges the gap between in vitro and in vivo systems in an extraordinary way, offering a high potential to reduce or replace animal experiments [51]. This model system can be used in translational medicine, for instance for drug testing before in vivo application in animal models or clinical studies. Nevertheless, several limitations of the microphysiological systems need to be considered. In addition to being expensive and complex to establish, organs-on-a-chip can only model certain aspects of tissue, not the entire organ. Further, the source material is limited and, most importantly, each type of tissue requires a specified medium matching its optimal conditions and, therefore, connecting different types of tissues is still challenging [53]. If the complexity of the model system needs to be further increased and/or standardized, another in vitro model would be 3D bioprinting. However, 3D bioprinting will not be discussed within this review and can be read elsewhere [54,55,56].

### 3.7. Animal Models

In vivo experiments are highly suited to investigate questions related to the whole organism or affecting several different aspects of it [57]. However, ethical concerns and challenges with the high experimental complexity need to be considered. In general, animal models are useful for analyzing in vivo responses in the actual microenvironment such as complex pathological processes, mechanisms of diseases, and tissue responses to insults. For example, mouse models have been useful in answering questions about epithelial repair and regeneration in the lung [58,59]. However, in vivo experiments have several limitations to be considered as well: they are expensive, in most cases highly time-consuming, and often show heterogenic results. When using mouse models in translational research, it is important to consider that the murine airway/lung anatomy and histology differ from humans and thus cannot fully resemble human physiology and diseases [60,61]. In contrast to humans, the conducting airways of mice directly transition from the bronchoalveolar duct junction into the alveolar space. Furthermore, the human bronchi have pseudostratified epithelia with basal, ciliated, and secretory cells, whereas murine bronchi have columnar epithelia including ciliated and club cells [61]. Considering this, pathological mechanisms may differ between mice and humans, as for example, the expressions of certain genes may differ between humans and mice [62]. Especially for the evaluation of drug candidates, animal experiments need to be appropriately designed, as small and large animals or rodents versus non-human primates offer different opportunities for this research. Most importantly, ethical issues need to be taken into account whilst working with animal models.

Organoids, despite not being the most complex in vitro model system, are one of the most developed complex systems as they represent a reasonable compromise between the level of complexity and necessary efforts and thus are a crucial tool for basic and translational research. They ideally close the gap between simple in vitro models and the in vivo situation. Therefore, the following chapters will focus on organoids as novel tools in lung research with high potential in translational and personalized medicine.

## 4. Human Lung Organoids

**Figure 3:**
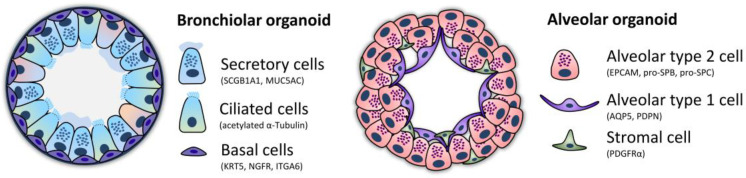
Schematic visualization of human bronchiolar and alveolar lung organoids and their cellular composition (adapted from Barkauskas et al. [8]).

As shortly introduced above, organoids are 3D, self-assembling cellular structures, composed of several different organ-specific cell types that execute their organ-specific cell function [47]. Organoids differ from spheroids by their more complex cellular composition, their capacity to self-renew and their need for extracellular scaffolds [63,64]. Lung organoids can be divided into different categories depending on the structures they represent alveolar organoids, bronchial organoids, bronchioalveolar organoids, and tracheospheres [8,28,65]. Bronchial lung organoids resemble the smaller conducting airways, hence basal, ciliated, and mucous-producing cells are mainly to be found in these bronchial lung organoids. In contrast, alveolar organoids resemble the structure of alveoli and consist, therefore, mainly of AT1 and AT2 cells (Figure 3). Table 1 summarizes information about different lung organoid types, their cellular composition, and their potential use.

Different kinds of lung organoids can be grown from a variety of primary cells, including embryonic stem cells, induced pluripotent stem cells or adult stem cells [31]. Each type of stem cell has different advantages and can be used to address different scientific questions. Embryonic stem cells and induced pluripotent stem cells find application in e.g., developmental research, whereas adult stem cells can be used to explore tissue repair or disease development in adult tissues. However, for embryonic lung bud tip organoids, embryonic tissue samples are needed, which are difficult to access [84,85]. Hein et al. [85] showed that their model of iPSC-derived lung bud tip organoids functionally and transcriptionally nicely represents the in vivo situation. Adult stem cells (e.g., certain kinds of basal cells in the case of bronchial structures) can be extracted easily from biopsy material obtained from healthy or diseased donors or via bronchial brushings. Further, only small amounts of donor material are necessary to establish an organoid culture, which can then be expanded and remains genetically stable [86,87]. Once put in culture, organoids can be maintained for a long period of time, at least up to one year [65,86]. Therefore, special culturing protocols have been established including splitting and diluting protocols. Splitting is the process of disrupting the 3D organoid structure into a single cell suspension, which could be in turn reseeded in the extracellular matrix where new 3D structures develop. Due to the proliferation and differentiation capabilities of the remaining stem cells as well as the self-assembling properties of epithelial cells, the organoids will regrow in the next passage. If the organoids will outgrow the extracellular matrix or are growing too dense, dilution protocols will become necessary. Here, intact organoids will be placed into a new extracellular matrix drop just at a lower density. It is also possible to reverse epithelial polarity and grow apical-out lung organoids which then can be more easily stimulated from the apical side [88,89]. A limitation of this model system is the lack of blood supply or additional cell types such as stroma and immune cells [87]. However, it is possible to co-culture organoids with other cell types, as it has been shown for instance with alveolar organoids co-cultured with fibroblasts or macrophages [19,90]. Another obstacle remaining to overcome whilst working with organoids is the difficulty of standardization as they are based on non-uniform patient-derived material. However, in comparison to the above-described model systems, organoids resemble a well-developed and highly complex cell culture model system used in lung research. 

In conclusion, for a great variety of scientific questions regarding lung diseases and (patho-)physiological processes in the lung, alveolar, or bronchial organoids can be applied. There are many protocols yet available to handle them in an easy way and to grow a long-term, nearly endless source of organoid culture [79,82,86,91].

## 5. Lung Organoids in Translational Research and Personalized Respiratory Medicine

Nowadays, due to emerging new technologies such as high-throughput assays, single-cell analysis, and bioinformatic tools, research has a greater potential to develop novel diagnostics and therapies for a wide range of diseases. However, finding suitable models for specific research questions remains often challenging. Model systems with a high transferability to the human in vivo situation is required for translational medicine in order to close the gap between fundamental research and clinical applications. A suitable solution is represented by the aforementioned complex 3D organoid model systems, as they consist of different functional organ-specific cell types with the ability to proliferate, differentiate and organize in a way very similar to in vivo processes. Miller et al. [92] depicted in a single-cell analysis the similarities of genes expressed in human (bud tip progenitor) lung organoids compared to the human fetal counterpart of the lung epithelium, proving the high transferability of in vitro organoid procedures to the human organism. Specifically, it was shown that basal cells within this organoid model have the ability to differentiate into multiple functional airway cells similar to the in vivo situation in fetal development [92]. Fetal model systems, however, are not useful for scientific questions concerning the pathogenesis of non-congenital airway diseases such as COPD or IPF. But also, for a better understanding of such disorders and the development of new therapeutic strategies for them, organoids may be of advantage, yet due to their high complexity and cellular diversity specific protocols for various experimental settings are required. Moreover, molecular methods were applied to further increase the potential of organoid model systems. For example, using fetal intestinal and alveolar lung organoids, Sun et al. [93] established an efficient genetic toolbox offering the possibility of CRISPR-mediated easy-tagging of desired gene loci within organoids.

Szabo et al. [94] suggested that amongst other methods lung organoids are suitable to be used in phenotype-based drug development platforms. In this approach, any given phenotype of a disease will be analyzed using an in vitro screening, thereby a potential therapy may be identified without a need to know each and every underlying mechanism of a specific phenotype. Further, organoids can be used for a high throughput drug screening on biosensor chips, including multi-well-linked systems that analyze a variety of organoids within the same culture environment. This offers great potential to assess drug efficacy as well as their side effects on different organs [95]. In the following subchapters, we discuss the application of organoids in the scope of translational research in different lung disease areas. 

### 5.1. Cystic Fibrosis

CF is an autosomal recessive disorder caused by mutations in the gene encoding for the CFTR channel resulting in its dysfunction. CFTR is essential for the physiological mucociliary clearance, hence its dysfunction results in mucus retention followed by airway inflammation and increased risk of airway infections [96]. Using single cell analysis, novel cell types present in the lung epithelium were identified, increasing the knowledge of CF disease pathomechanisms. For instance, by analyzing murine epithelial tracheal cells and human epithelial cells after ALI cultivation, Plasschaert et al. [97] identified *Foxi1^+^* pulmonary ionocytes specifically expressing CFTR, which might be involved in the pathomechanisms of CF [97]. Furthermore, the main features of CF could be modeled using human lung organoids derived from the patient’s source material or by inducing a certain mutation in vitro. For example, Schwank et al. [98] used human intestinal organoids to analyze and modulate the mechanisms of CF. They corrected by CRISPR/Cas9-mediated gene editing the mutated CFTR locus in intestinal stem cells, which restored their cellular function [98]. To evaluate effects in the lung, McCauley et al. [99] established a model of CF representing lung organoids based on patient-derived iPSCs. Such organoids are already used for drug screening or in fundamental research of CF [99]. Forskolin is a diterpene, which is used as a stimulating agent in a swelling test, as it induces a CFTR-dependent swelling of epithelial cells in organoids [100]. McCauley et al. [99] were able to rescue the swelling function of CF-patient-derived organoids by gene editing and Hirai et al. [101] showed that human lung organoids derived from pluripotent stem cells modelling CF can be used for drug testing of CFTR modulators by performing a forskolin-stimulation swelling test. Upon stimulation, the lung organoids responded in a mutation-dependent manner. 

Thus, organoids add from our point of view several great advantages to this research area, especially since CF is still not curable.

### 5.2. Idiopathic Pulmonary Fibrosis

Another common lung disease is IPF, a severe chronic disorder associated with several risk factors leading to the remodeling processes of the lung epithelium with increased production of extracellular matrix [102,103]. Jaeger et al. [104] were able to establish an organoid model based on co-cultures of airway basal cells and fibroblasts derived from IPF patients, showing increased fibroblast proliferation and intensified bronchosphere formation in the IPF organoid model compared to non-IPF organoids. Recently, Chen et al. [80] and Strikoudis et al. [83] implemented fibrotic formations in hPSC-derived organoids, by inducing disease-causing mutations in the Hermansky–Pudlak Syndrome (HPS) genes via CRISPR/Cas9. HPS is a rare genetic disorder characterized by albinism, immunodeficiency, bleeding diathesis, and pulmonary fibrosis [105]. For instance, Strikoudis et al. [83] suggested targeting IL-11 as a new therapeutic strategy, since high levels of IL-11 expression induced fibrosis, while its depletion impeded fibrosis in organoids [83]. Valdoz et al. [106] established another fibrotic alveolar lung organoid model by bleomycin-induced fibrosis in a combined culture of epithelial (A549), endothelial and fibroblast cell lines grew as organoids. Upon this induction, organoids showed increased fibroblast proliferation and lumen reduction, which could be partially rescued by the anti-fibrotic treatment with the Rho-kinase inhibitor fasudil [106]. Further, by using organoids Kathiriya et al. [107] showed the transdifferentiation of human alveolar epithelial type two cells (hAEC2) into metaplastic KRT5^+^ basal cells under the influence of for instance TGF-β signaling or *CTHRCI^hi^* pro-fibrotic mesenchyme. As hAEC2 with basal cell characteristics are found in IPF lungs, driving this research on organoids forward could further advance insights into IPF pathogenesis. In our assessment, organoids as complex in vitro model systems have a high potential to generate new insights in IPF pathogeneses and hence the development of therapeutic strategies, as cell–cell interactions and co-culturing with mesenchymal cells is suitable to mirror the in vivo situation of fibrotic lungs.

### 5.3. Chronic Obstructive Lung Diseases—COPD and Asthma

COPD is a common disease worldwide, affecting primarily older patients with multimorbidity after being exposed to several risk factors such as cigarette smoke. COPD is an inflammatory airway disease, manifested by chronic bronchitis and emphysema based on various underlying pathomechanisms [108]. This etiopathogenetic diversity results in a need for complex in vitro lung model systems that ideally represent different types of disease. Basil et al. [109] revealed by using human alveolar lung organoids a respiratory airway secretory (RAS) cell population as progenitors of alveolar type two cells. This SCGB1A1^+^ and SCGB3A2^+^ cell population shows a modified transcriptome profile in patients with COPD leading to altered AT2 cell conditions. Further, transitioning of RAS into AT2 cells seems to be affected by smoke injury as a pack-year dependent increase in SCGB3A2^+^ and LAMP3^+^ cell populations was shown. Song et al. [110] tested a D-dopachrome tautomerase (DDT) treatment using human and murine alveolar lung organoids modeling COPD, resulting in increased organoid growth following application. Therefore, they suggested DDT as a basis for the development of further therapeutic strategies for the treatment of COPD but also mentioned considering the pro-tumorigenic properties of this compound [110]. Despite having the potential to accurately mimic major features of COPD, human lung organoids are not commonly used in COPD research so far. To the best of our knowledge, the same is the case for bronchial asthma, even though bronchial lung organoids could adequately mimic the main disease properties. Goblet cell metaplasia associated with increased mucus production is a crucial component of obstructive airway diseases such as COPD and bronchial asthma [111,112]. Danahay et al. [68] were able to induce a goblet cell metaplasia phenotype in human bronchiospheres by the application of IL-13 and IL-17A. By identifying NOTCH2 as an important driver of a goblet cell metaplasia phenotype, NOTCH2 was proposed to be tested as a new therapeutic target in obstructive airway diseases [68,113]. COPD research is still often performed based on ALI cultures since smoke exposure is easily modeled in this system. In our assessment, however, we see organoids here as well on an upward trend as for instance apical-out organoids are currently in development. Chiu et al. [89] used these apical-out lung organoids to model infection with the severe acute respiratory syndrome coronavirus 2 (SARS-CoV2) but exposures with other (non-infectious) substances are as well conceivable. 

### 5.4. Infectious Diseases

The COVID-19 pandemic pointed again to the persistent importance of research in infectious airway diseases. It has been demonstrated that lung organoids have considerable potential for application in translational research to study infectious diseases. Chen et al. [80] and Porotto et al. [114] demonstrated that hPSC-derived organoids infected with respiratory syncytial virus (RSV) exhibit features of an infected airway epithelium, including swelling and detachment of cells. Human parainfluenza virus type 3 (HPIV3) infected organoids also showed reduced integrity [80,114]. In addition, Zhou et al. [115] established a bronchial lung organoid model to investigate the infectivity of different influenza virus strains, which might be useful in future surveillance of evolving viral types. Further, organoid models mimicking infections with several other viruses, e.g., enterovirus 71, or those mimicking bacterial infections have also been established [116,117]. Shen et al. [117] analyzed host–microbe interactions using murine lung organoids and a recombinant flagellar protein. Furthermore, Hong and Seo [118] discussed the possible use of organoids for biotechnical virus production (e.g., for vaccine development) for strains that are difficult to cultivate by conventional procedures. As the COVID-19 pandemic showed effective ways of antiviral drug development are needed for which organoids may represent a suitable component in preclinical research. For instance, Katsura et al. [119] used alveospheres to analyze SARS-CoV-2-mediated interferon responses, revealing thereby that a low-dose interferon pre-treatment could reduce viral replication. In conclusion, since lung organoids mirror host-pathogen interactions at a high organizational level, they have a strong potential to provide more insights into pathomechanisms driving infectious airway diseases and to develop new therapeutic strategies based on this knowledge.

### 5.5. Oncology

Another possibility to use organoids in personalized medicine is the generation of tumor organoids from non-small cell lung carcinoma (NSCLC) patients [120,121]. NSCLC patient-derived tumor organoids were established, which preserved tumorigenicity, malignancy, and mutations in comparison to the original tumor as shown by whole-exome and RNA sequencing. A major problem with tumor organoids remains that they tend to get overgrown by normal/healthy organoids [120]. However, lung tumor organoids might be applicable to screen drugs or for the search for new biomarkers. Specifically, tumor spheroids can be used as ex vivo systems to develop combinatorial therapies in a personalized way. This was shown by Jenkins et al. [122] analyzing mouse-derived and patient-derived organotypic tumor spheroids including autologous immune and stroma cells to analyze the effects of a combined immune checkpoint blockade with PD-1 and TANK-binding kinase 1/I-kappa-B kinase epsilon (TBK1/IKKε) inhibitors, so-called compound 1 (Cmpd1). Further, they demonstrated that cytokines secreted by the patient-derived tumor organoids could be used to predict responses to a PD1 blockade. This approach might be used as a functional assay to predict individual responses to potential immunotherapies in oncology [122]. Likewise, Veelken et al. [123] provided a workflow for high-throughput analysis of invasive melanoma tumor spheroids. They showed that viability tests enable large-scale screenings of therapy responses. However, due to the tumor heterogeneity additional single-cell analyses are needed, without them, it might be difficult to evaluate the therapeutic efficacy based on the organoid model [123].

## 6. Future Perspectives for Lung Transplantation and Gene Therapy

To further expand the potential of personalized medicine, molecular methods can not only be used to induce mutations in organoids for specific drug screenings or fundamental research but can also be applied to correct altered genes as a methodological contribution to the rapidly developing field of gene therapy. Leibel et al. [124] demonstrated the potential of patient-specific iPSC-derived lung organoids to develop new approaches to target and correct genetically caused lung diseases. For example, they were able to rescue a surfactant secretion deficiency in such organoids via viral gene therapy [124]. This is inevitably followed by the question of the transplantability of organoids. As depicted by Tian et al. [125], the main problem of in vivo transplantation of organoids is the absence of vessels to transport nutrients or waste. Thus, despite huge potential, there are still some obstacles to overcome. It is promising that there are protocols available, by which organoids may be hindered from differentiating into unwanted cell types thus eliminating concerns about the formation of teratomas after transplantation [125]. Besides, in different studies with human intestinal organoids transplanted into mice, these organoids showed an adult-like pattern of gene expression compared to organoids cultured in vitro [126,127]. Miller et al. [81] successfully transplanted hPSC-derived lung organoids into injured mouse lungs; however, it remains to be evaluated whether these organoids are functionally and stably integrated over a long time [81]. Another approach to transplanting lung organoids has been applied by Trecartin et al. [128]. Murine and human lung organoids were transplanted into immunodeficient mice to develop tissue-engineered lungs. This procedure offers a future possibility for lung regeneration and repair to improve survival following severe lung diseases [128]. However, considering that immunocompromised mice are not the optimal proxy for humans, we might still be far from applying this approach as treatment in the clinics.

Different approaches have been performed for reseeding decellularized lung scaffolds populated with autologous cells followed by the transplantation of the resulting organoid structures. Hayes et al. [129] suggested that about 60 million cells would be needed to regenerate a human lung. The transplantation of bioengineered lungs was conducted for example in rats [130,131]. Ren et al. [132] established a method to repopulate the vascular compartment of decellularized rat and human lung scaffolds. For the rat lung scaffold, they achieved coverage with endothelial cells of approx. 75%. After transplantation, these recellularized endothelial cells constituted a functional vascular barrier for three days. In a further approach, they reseeded a human lung lobe with human iPSCs. Even though the human lung lobe did not achieve that high numbers of coverage, generally this might be a way to engineer lungs in vitro to replace donor lungs that are frequently missing for transplantation [132]. The review by Wancyk et al. [133] provides a deeper understanding of how single-cell or multiomics approaches are further shaping modern developments in bioengineered lung technology.

**Figure 4:**
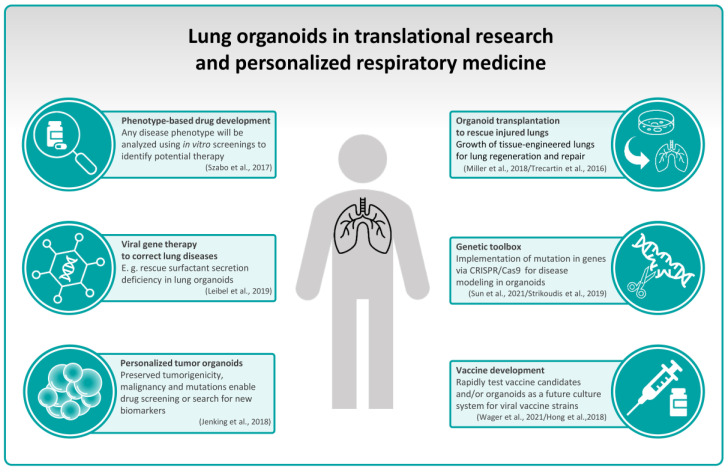
Lung organoids have a huge potential in translational and personalized medicine. Applications and future possible usages are depicted [81,83,93,94,118,122,124,128,134].

In the future, due to the development of patient-based model systems that mimic in vivo conditions up to a high level, patients will receive individual therapies that are best suited to their specific needs and conditions (Figure 4).

## 7. Conclusions

As this review demonstrates, human lung organoids present a ground-breaking method for research on lung physiology and pathology, even though the method is still in its infancy. There is a number of established protocols for the expansion and culture of human lung organoids available. Whereas current protocols focus on modelling compartments of the respiratory system and co-culture of different lung cell types as functional organoids, the next step will be the further development of advanced organoids additionally combining immunological settings with perfusable vasculature for an even more accurate representation of the branching architecture of the respiratory system [106,135]. In the future, human lung organoid models will be perfected to incorporate the benefits of cell line and ALI cultures, thus becoming cheaper and easier to stimulate apically. Human lung organoids, especially those cultured from patient tissue samples, offer a unique opportunity which cannot be achieved by single-cell line cultures. Specifically, they are able to mirror patients’ individual diseases and personal genetics and molecular biology. As modern medicine evolves towards personalized therapies, patient-specific lung organoids will become a testing ground for biomarkers and drug development. In addition, individual therapy responses and resistance mechanisms may be determined on the basis of which an optimal individual treatment strategy may be established. The need for efficient respiratory disease models is extraordinarily high, especially in the aftermath of the global respiratory pandemic. Lung organoids have exponentially expanded the possibilities of in vitro respiratory research using human-derived biomaterials.

## Figures and Tables

**Table 1:** Origin and composition of different lung organoid types with exemplary application of these organoids as model systems in translational research.

Organoid Type	Description/Origin	Cell Types & Markers	Modeling	Sources
**Tracheospheres**	Spheroids grown from tracheal stem cells	**Basal cells**: p63^+^, KRT5^+^, KRT14^+^, NGFR^+^, ITGA6^+^ **Secretory cells**: KRT8^+^ Goblet cells: MUC5AC^+^ **Ciliated cells**: α-tubulin^+^	Self-renewal & differentiation processes Inflammatory goblet cell metaplasia	**Human and murine**: Rock et al., 2009 [66] **Murine**: Tadokoro et al., 2014 [67]
**Bronchiospheres**/ **Bronchial** **organoids**	Derived from progenitor cells of bronchi: mainly basal cells, also club cells AT2 cells co-cultured with adult human lung mesenchymal cells	**Basal cells**: p63^+^, KRT5^+^, NGFR^+^, ITGA6^+^, PDPN^+^, KRT14^+^ **Club cells**: SCGB1A1^+^, SCGB3A2^+^, SPLUNC1^+^ **Goblet cells**: MUC5AC^+^ **Ciliated cells**: acetylated tubulin^+^, α-tubulin^+^, FOXJ1^+^ **Neuroendocrine cells**: CGRP^+^ **Pulmonary ionocytes**: FOXI1^+^ **General epithelial cell markers**: KRT8^+^, E-cadherin^+^	Goblet cell metaplasia Bronchial asthma COPD Cystic fibrosis	**Human**: Danahay et al., 2015 [68] Ekanger et al., 2022 [69] Hild and Jaffe, 2016 [6] **Murine**: Kathiriya et al., 2020 [70] Lee et al., 2017 [71] Rabata et al., 2020 [72] **Human and murine**: Rock et al., 2009 [66]
**Alveolar** **organoids**	From alveolar progenitor cells: AT2 cells	**AT1**: AQP5^+^, PDPN^+^, HTI-56^+^; flat form **AT2**: EPCAM^+^, HTII-280^+^, pro-SPB^+^ and pro-SPC^+^; cuboidal form, stem cell capacity	Injury repair and idiopathic pulmonary fibrosis Screening of small inhibitory molecules Crosstalk with co-cultured cells Screening of small inhibitory molecules Infectious diseases Alveospheres: scRNAseq analysis, differentiation of alveolar cells	**Human**: Alysandratos et al., 2023 [73] Choi et al., 2020 [74] Ekanger et al., 2022 [69] Lamers et al., 2021 [75] Youk et al., 2020 [76] **Murine**: Kobayashi et al., 2020 [77] Lee et al., 2017 [71] Rabata et al., 2020 [72] Sun et al., 2019 [78] **Human and murine**: Barkauskas et al., 2013 [19]
**Bronchioalveolar organoids**	Lung tissue samples: CHIR99021-induced SCGBb1A1^+^ cells co-culture with LGR6^+^ cells	Bronchial and alveolar features	Injury repair	**Human**: Hoareau et al., 2021 [79] **Murine**: Lee et al., 2017 [71]
**Lung bud** **organoids**	hPSCs (of mesoderm and pulmonary endoderm) develop into airway organoids	See bronchial and alveolar organoids Different growth factors drive differentiation of progenitor cells into lung epithelial cells	RSV infection Fibrosis	**Human**: Chen et al., 2017 [80] Miller et al., 2018 [81] Miller et al., 2019 [82] Strikoudis et al., 2019 [83]

AT1 = alveolar cell type 1, AT2 = alveolar cell type 2, AQP5 = aquaporin 5, CGRP = calcitonin gene-related peptide, COPD = chronic obstructive pulmonary disease, EPCAM = epithelial cell adhesion molecule, FOXJ1 = forkhead box J1, hPSCs = human pluripotent stem cells, ITGA6 = integrin subunit alpha 6, KRT = keratin, LGR6 = leucine-rich repeat containing G protein-coupled receptor 6, MUC5AC = mucin 5AC, NGFR = nerve growth factor receptor, PDPN = podoplanin, SPB/SPC = surfactant protein B/Cp63 = tumor protein p63, RSV = respiratory syncytial virus, SCGB1A1 = secretoglobin family 1A member 1, SCGB3A2 = secretoglobin family 3A member 2, scRNAseq = single-cell RNA sequencing. SPLUNC1 = short palate lung and nasal epithelial clone 1, also known as BPIFA1 = BPI fold containing family A member 1. Remark: all markers are annotated as human genes for unification.
